# 

**Published:** 1865-03

**Authors:** 


### ILLINOIS STATE MEDICAL SOCIETY.

The regular Annual Meeting of the Illinois State Medical Society will be held in the city of Bloomington, commencing on the *first Tuesday* in May, 1865. Each local Medical Society is entitled to one delegate for every ten of its members. Each regular Medical College and each Hospital is entitled to two delegates. We hope to see a full attendance, and enjoy a profitable meeting.

N. S. Davis,

*Permanent Secretary.*

Chicago, Feb. 10th, 1865.

### REMOVAL.

☞ The Editorial and Private Office of DRS. MILLER and INGALS will hereafter be at No. 190 South Clark St., between Monroe and Adams Sts.

☞ Business letters to the Journal should be addressed to Drs. Miller & Ingals, Chicago, Ills., P. O. Drawer 5787. When money is received a receipt will be returned with the next number of the Journal, and subscribers failing to get such receipt will confer a favor by giving us notice of any omission.

